# Genomic analysis of community-associated methicillin-resistant *Staphylococcus aureus* (CA-MRSA) causing infections in children—a Spanish multicenter study

**DOI:** 10.3389/fmicb.2025.1534840

**Published:** 2025-05-09

**Authors:** Silvia García-Cobos, Natalia Seco Alberca, Blanca Bravo-Queipo-de-Llano, Verónica Casquero-García, Eva Ramírez de Arellano, Cristina Calvo, Guillermo Ruíz-Carrascoso, Iker Falces-Romero, Nieves Larrosa Escartín, Belén Viñado-Perez, Miguel Ángel Martínez-López, Susana Melendo Pérez, Enrique Ruíz de Gopegui, Santiago Pérez Vázquez, Jaime Carrasco-Colom, Belén Aracil García, María Pérez-Vázquez, Ana Méndez-Echevarría, Jesús Oteo Iglesias

**Affiliations:** ^1^Laboratorio de Referencia e Investigación en Resistencia a Antibióticos e Infecciones Relacionadas con la Asistencia Sanitaria, Centro Nacional de Microbiología, Instituto de Salud Carlos III, Majadahonda, Madrid, Spain; ^2^CIBER de Enfermedades Infecciosas, Instituto de Salud Carlos III, Madrid, Spain; ^3^Servicio de Pediatría y Enfermedades Infecciosas, Hospital Universitario La Paz, Fundación IdiPaz, Madrid, Spain; Universidad Autónoma de Madrid, Red de Investigación Traslación en Infectología Pediátrica (RITIP), Madrid, Spain; ^4^Servicio de Microbiología, Hospital Universitario La Paz, Fundación IdiPaz, Madrid, Spain; ^5^Servicio de Microbiología, Hospital Universitario Vall d’Hebron, Barcelona, Spain; ^6^Vall d'Hebron Institut de Recerca (VHIR), Barcelona, Spain; ^7^Servicio de Pediatría, Hospital Universitario Vall d’Hebron, Barcelona, Spain; ^8^Servicio de Microbiología, Hospital Universitario Son Espases, Mallorca, Spain; ^9^Servicio de Pediatría, Hospital Universitario Son Espases, Mallorca, Spain

**Keywords:** *Staphylococcus aureus*, community-associated infections, CA-MRSA, children, skin and soft tissue infections

## Abstract

**Objectives:**

*Staphylococcus aureus* is one of the most common human pathogens causing skin and soft tissue infections (SSTIs) among children. This study investigated the molecular traits of community-associated methicillin-resistant *S. aureus* (CA-MRSA) isolates causing infections in children in Spain.

**Methods:**

Antibiotic susceptibility testing and whole-genome sequencing were performed in 98 CA-MRSA isolates (4.2 median age, 52% males). The phylogenetic relationship, antibiotic resistance, virulence, and plasmid replicon genes content were investigated.

**Results:**

Resistance rates were found as follows: Erythromycin, 42.9%, which could be explained due to the presence of *erm(C)*, *mph(C)*, and *msr(A)* genes; tobramycin, 27.5%, which could be explained due to the presence of *aac(6′)-Ie/aph(2″)-Ia* and *aadD1* genes; tetracycline, 25.5%, which could be explained mainly due to the presence of *tet(K)* genes; levofloxacin and moxifloxacin, 19.4%, which could be explained primarily due to the mutations in *gyrA* and *parC* genes; and gentamicin, 15.3%, which could be explained due to the presence of *aac(6′)-Ie/aph(2″)-Ia* gene. The most prevalent lineage was ST8-IVc and t008. Most isolates were genetically diverse, except for three groups of isolates from the same hospital and one group of isolates from different hospitals. These had less than or equal to 5 allele differences by core-genome multilocus sequence typing (cgMLST) analysis or 0–6 core single-nucleotide polymorphisms (SNPs) by core-genome SNP-based analysis. Phage-encoded Panton–Valentine leukocidin (PVL) genes were found in 75.5% of the isolates. Other common virulence genes were related to adhesion (*capA* and *capP*), lipid degradation (*geh*), hemolysis (*hlb*, *hld*, *hlgABC*, and *hly/hla*), and tissue destruction (*sspAB*).

**Conclusion:**

This study observed a high genetic diversity among CA-MRSA isolates causing community-acquired infections in children in Spain, with ST8-IVc as the most prevalent lineage. Nevertheless, genetic relatedness of some isolates from the same as well as different hospitals suggests the dissemination of CA-MRSA among children by contact.

## 1 Introduction

*Staphylococcus aureus* is one of the most common human pathogens, generally associated with skin and soft tissue infections (SSTIs) due to its affinity for skin and mucous membranes, and may cause bacteremia and other persistent chronic diseases, leading to high morbidity and mortality in children (Tong et al., 2015; Chalmers and Wylam, 2020). In children, *S. aureus* is not only one of the leading pathogens causing SSTIs but also a common cause of bloodstream infection (BSI), commonly as a secondary infection or being associated with more virulent *S. aureus* strains, such as Panton–Valentine leukocidin (PVL) producing strains (Kaplan, 2006; Kalu et al., 2022).

*S. aureus* is continually evolving and progressively acquiring resistance mechanisms to the majority of antibiotics (Cassat and Thomsen, 2021; Pantosti et al., 2007). After the spread of penicillin-resistant *S. aureus* strains worldwide, the semi-synthetic penicillin, methicillin, was introduced in clinical practice. Unfortunately, strains resistant not only to methicillin but to the entire class of *β*-lactam antibiotics emerged shortly thereafter (Chambers and DeLeo, 2009), becoming one of the primary threats of antibiotic resistance worldwide, as recognized by the World Health Organization (WHO) (Tacconelli et al., 2018). Methicillin resistance in *S. aureus* is due to the production of an additional penicillin-binding protein (PBP), designated PBP2a, which is not inhibited by *β*-lactams. PBP2a is codified by the *mecA* gene and transported in a 30–60 kb element, denominated staphylococcal chromosomal cassette (SCC)*mec* (Pantosti et al., 2007). In addition, homologs of *mecA*, such as *mecC*, have been described. SCC*mec* elements are classified into types and subtypes. SCC*mec* types are based on the combination of the *mec* gene complex and the *ccr* gene complex types, with fourteen SCC*mec* types up to date, described using Roman numerals (Uehara, 2022). The classification of SCC*mec* subtypes is based on J (“junkyard”) regions, which may carry additional antimicrobial resistance determinants and may be located in different positions (Uehara, 2022).

In Spain, methicillin-resistant *S. aureus* (MRSA) prevalence in blood samples ranged between 23 and 25.8% in the last years, according to the data reported by the European Antimicrobial Resistance Surveillance Network (EARS-Net) (The European Committee on Antimicrobial Susceptibility Testing, 2023). MRSA isolates are commonly associated with hospitalization (HA-MRSA). However, in the mid-1990s, clinically and genetically distinct MRSA strains emerged, affecting healthy children and adults lacking traditional risk factors (Gorak et al., 1999; Elston, 2007). These strains, called community-associated MRSA (CA-MRSA), were mostly related to SSTIs and could be more virulent, often producing PVL cytotoxin (Boyle-Vavra and Daum, 2007; DeLeo et al., 2010). CA-MRSA isolates are typically associated with SCC*mec* types IV and V, which are usually composed solely of methicillin-resistant genes and thus are smaller SCC*mec* elements, being more susceptible to non-*β*-lactam antibiotics, including several orally available agents, compared to HA-MRSA (Herold, 1998; David and Daum, 2010).

Different CA-MRSA lineages have been described worldwide, with sequence type 80 (ST80) usually carrying an SCC*mec* type IV as the most common clone in Europe, North Africa, and the Middle East (Mairi et al., 2020). Other common lineages described are ST30-IV (Southwest Pacific clone) in East Asia and Oceania, ST1-IV (USA400 clone) and ST8-IVa (USA300 clone) in the United States (Mairi et al., 2020). In Spain, the most common clone is the PVL-positive ST8-IVc, while ST30-IVc, ST80-IVc, and ST5-IVc are less prevalent (Vindel et al., 2014).

The prevalence of CA-MRSA in the pediatric population varies among countries. However, large-scale multicenter studies are limited, and the majority of the studies did not provide detailed molecular-level information. A retrospective study in a hospital in North Carolina reported a 75.9% prevalence of CA-MRSA in children over 7.5 months in 2006 (Shapiro et al., 2009). A study including 39 pediatric hospitals in the USA observed a decrease in MRSA infections by 52% from 2009 to 2016. However, the study design did not allow for distinguishing between hospital-onset vs. community-onset infections (Spaulding et al., 2018). A retrospective observational study of a 10-year review in a hospital in Taiwan reported 56.8% (363/639) of CA-MRSA among children (Yueh et al., 2022). The prevalence of CA-MRSA colonizations/infections was 62% (31/50) in Birmingham, UK, data from three hospitals with pediatric services (Adedeji et al., 2007). A cross-sectional study in Paraguay reported an increase of CA-MRSA prevalence in children from 21% (24/113) between 2009 and 2010 to 54% (91/168) between 2012 and 2013, with a dominant clone, ST30-IV-t019 (Rodriguez et al., 2020).

In Spain, CA-MRSA infections in children were reported for the first time in 2006, these isolates were PVL positive and had the SCC*mec*-IV (Broseta et al., 2006). Another study in 2007 in an emergency department in Madrid, Spain, found 13.2% (7/53) cases of CA-MRSA causing SSTI. PVL-positive isolates were more likely to cause severe local disease and belonged to ST8 (Daskalaki et al., 2010). A recent multicenter study involving children from community settings across Spain (the COSACO study—Colonization by *S. aureus* in the Community) showed a prevalence of MRSA colonization at 1.4%, with no strains producing PVL and associated with rural settings (Del Rosal et al., 2020).

Nevertheless, up-to-date data regarding CA-MRSA infections in children in Spain are scarce, and still, little information is available on the genetic traits of these strains. This study aimed to characterize CA-MRSA isolates causing pediatric infections in Spain using whole-genome sequencing (WGS) and to move forward to the implementation of WGS in the surveillance of MRSA infections following the roadmap set by the European Centre for Disease Prevention and Control (ECDC) (European Centre for Disease Prevention and Control, 2019). This study gathers genomic information about (i) genetic relatedness of CA-MRSA isolates causing pediatric infections in Spain using WGS-based typing methods, including *spa*-type, multilocus sequencing typing (MLST), core-genome multilocus sequence typing (cgMLST), and core-genome single-nucleotide polymorphism (SNP)-based analysis; (ii) antibiotic resistance mechanisms; and (iii) key virulence factors.

## 2 Materials and methods

### 2.1 Isolate collection

A total of 98 community-associated methicillin-resistant *S. aureus* (CA-MRSA) isolates causing infections in children, one isolate per patient, were collected via three reference and large Spanish tertiary hospitals between 2018 and 2022 (4.2 median age, 52% males). Isolates obtained 48 h after admission or from 3 months previously hospitalized patients were excluded to ensure community origin. Medical records from children with a positive culture of MRSA, obtained from abscesses, biopsies, blood cultures, bronchoalveolar lavages, and/or biological fluids (joint, pleural, and cerebrospinal fluid), were reviewed and included. In addition, SSTIs and conjunctivitis cases were included based on a positive culture of MRSA from the infection site, clinical symptoms, and absence of polymicrobial infections. Isolates interpreted as colonization or contamination were excluded. Majority of the isolates, 84% (83/98), were isolated from SSTIs, 10% (10/98) from respiratory infections, 2% (2/98) from bone infections, 1% (1/98) from urine infection, 1% (1/98) from conjunctivitis, and 1% (1/98) from primary blood infections.

### 2.2 Antibiotic susceptibility testing

Antibiotic susceptibility testing (AST) was performed using the broth microdilution method (EUSTAPF Sensititre TM panels, Thermo Fisher Scientific, Waltham, Massachusetts, USA) and interpreted following EUCAST v12.0 clinical breakpoints (The European Committee on Antimicrobial Susceptibility Testing, 2023). The following antibiotics were tested: ceftaroline, cefoxitin, clindamycin—along with D-test for inducible clindamycin resistance—daptomycin, erythromycin, fusidate, gentamicin, levofloxacin, linezolid, moxifloxacin, rifampin, telavancin with tween, teicoplanin, tetracycline, trimethoprim/sulfamethoxazol, tobramycin, and vancomycin.

### 2.3 Whole-genome short-read sequencing and sequence analysis

#### 2.3.1 Library preparation and whole-genome sequencing (WGS)

WGS was performed after DNA extraction using the DNeasy UltraClean Microbial Kit (Qiagen, Hilden, Alemania) (Qiagen, n.d.) and subsequent library preparation using DNA Nextera XT (Illumina, San Diego, CA, USA) (Illumina, n.d.). Libraries were sequenced using the Illumina NexSeq 6,000 Sequencing system with 150-base paired-end reads.

#### 2.3.2 Contamination screening

Raw reads were analyzed using Mash Screen v2.2.2, comparing a subset of 1,000 k-mers per isolate against all the National Center for Biotechnology Information (NCBI) RefSeq genomes (release 88) (O’Leary et al., 2016) to confirm the species identification, detect contamination with other species, and select a standard reference for an efficient variant call (Ondov et al., 2019).

#### 2.3.3 Quality trimming of short reads and draft genome assemblies

Short reads were quality trimmed and *de novo* assembled using Unicycler v.0.4.8^1^ (Wick et al., 2017). Genome assembly quality was assessed using QUAST v5.0.2^2^ (Gurevich et al., 2013).

#### 2.3.4 Sequence type (ST)-MLST and core-genome multilocus sequence typing (cgMLST) analysis

Genome assemblies were uploaded to Ridom SeqSphere+ v9.0 commercial software (Münster, Germany)^3^ (Jünemann et al., 2013) for a gene-by-gene comparison using a cgMLST scheme of 1,861 targets^4^ and to obtain ST-MLST (Enright et al., 2000; PubMLST, n.d.) and *spa*-types. We applied a threshold of less than or equal to 5 allele differences to indicate genetic relatedness. This threshold is based on a previous study in which the authors assessed the genomic variation rate in MRSA isolates obtained from long-term carriers. The cgMLST analysis using Ridom SeqSphere+ showed a median of 5.0 allele variants/year, and the authors concluded an estimated genomic variation rate of 2.0–5.8 genetic events per year (without recombination) (Lagos et al., 2022).

#### 2.3.5 Core-genome SNP-based phylogenetic analysis

The genome NZ_CP026076.1 served as a reference based on Mash Screen results. A core-genome alignment of 66,472 bp SNP sites was obtained using Snippy v4.6.0.^5^ This consensus SNP-sites alignment was used to build a maximum-likelihood tree with RaxML-NG v1.0.3.^6^ A general time-reversible model with gamma correction among-site rate variation (GTR + G4) was used. The support for the nodes was assessed using 100 bootstrap replicates. The phylogenetic tree was visualized with iTol (Letunic and Bork, 2021).

#### 2.3.6 Antibiotic resistance genes, virulence genes, and plasmid replicon genes

SSC*mec* typing was done using *staphopia-sccmec* v1.0.0 from *staph-typer* subworkflow^7^ (Petit and Read, 2018) included in Bactopia tools (Petit and Read, 2020) and SCCmecFinder 1.2 web-based tool from the Center for Genomic Epidemiology^8^ (Kaya et al., 2018). *Agr* typing was done using *agrvate* v1.0.2, also included in the *staph-typer* subworkflow. The AMRFinderPlus tool included Ridom SeqSphere+ v9.0, which was used to analyze point mutations related to fluoroquinolone resistance. Assembled genomes were screened using Abricate 1.0.1^9^ and the following databases: ResFinder for identifying acquired antimicrobial resistance genes^10^ (Camacho et al., 2009; Bortolaia et al., 2020), virulence factor database (VFDB) for identifying virulence factors,^11^ and PlasmidFinder for plasmid replicon genes^12^ (Camacho et al., 2009; Carattoli et al., 2014) (downloaded last on: 27 March 2023). Isolates with a resistance phenotype but no antibiotic resistance genes (ARGs) found when screening assemblies were additionally analyzed using ARIBA 2.14.6 based on trimmed reads screening,^13^ with the following databases: ResFinder, ARG-ANNOT,^14^ and the Comprehensive Antibiotic Resistance Database (CARD).^15^ Other virulence factors not included in VFDB, such as copper and mercury resistance genes (COMER) and arginine catabolic mobile element (ACME), were screened using Abricate 1.0.1 (Supplementary Table S1). RFPlasmid v0.0.18 was used to predict chromosomal and plasmid contigs^16^ (Van Der Graaf-van et al., 2021). RFPlasmid results and Abricate results for antibiotic resistance genes, virulence factors, and plasmid replicon genes were combined to elucidate the predicted location of these genes.

#### 2.3.7 Prophage detection

Prophage sequence identification was done using DBSCAN-SWA, a command-line software tool developed to predict prophage regions in bacterial genomes^17^ (Gan et al., 2022).

### 2.4 Genome comparative analysis with MRSA isolates from nasal colonization in Spanish children

For comparison purposes, 19 MRSA isolates on nasal colonization in Spanish children (Del Rosal et al., 2020; Román et al., 2021) from a previous study were additionally sequenced, using the same methodology for DNA extraction, library preparation, and whole-genome short-read sequencing as described above. Previous characterization showed that these isolates belonged to the following sequence types: ST5 (*n* = 5, 26.3%), ST30 (*n* = 4, 21.1%), ST125 (*n* = 3, 15.8%), ST22 (*n* = 2, 10.5%), ST72 (*n* = 2, 10.5%), ST6 (*n* = 1, 5.3%), ST34 (*n* = 1, 5.3%), and ST6690 (*n* = 1, 5.3%). The *spa*-types were t002 (*n* = 6, 31.6%), t012 (*n* = 2, 10.5%), t067 (*n* = 2, 10.5%), and t021, t223, t790, t1507, t4100, t4407, t2532, and t20101 with one isolate (5.3%) (Román et al., 2021). Antibiotic susceptibility testing was also performed in these isolates using the broth microdilution method (EUSTAPF Sensititre TM panels, Thermo Fisher Scientific, USA).

## 3 Results

### 3.1 Antibiotic resistance profile

The highest resistance rate of CA-MRSA isolates was against erythromycin (42.9%), followed by tobramycin (27.5%), tetracycline (25.5%), fluoroquinolones—levofloxacin and moxifloxacin—(19.4%), and gentamicin (15.3%). Some CA-MRSA isolates also had resistance to fusidate (8.2%), trimethoprim/sulfamethoxazole (4.1%), rifampicin (3.1%) and clindamycin constitutive (2.0%) or inducible (19.4%). All isolates were susceptible to ceftaroline, daptomycin, linezolid, teicoplanin, telavancin, and vancomycin (Table 1). The most potent antibiotics were ceftaroline, clindamycin, daptomycin, fusidate, linezolid, rifampin, teicoplanin, telavancin with tween, trimethoprim/sulfamethoxazole and vancomycin, with MIC_90_ of ≤0.5, ≤0.12, ≤0.5, 1.0, ≤2.0, ≤0.03, ≤1.0, 0.06, 1.0, and 1.0 mg/L, respectively (Table 1).

**Table 1:** Antibiotic susceptibility testing results for CA-MRSA isolates causing pediatric infections in Spain.

Antibiotic (dilution range mg/L)	Resistance (%)	MIC_50_ (mg/L)	MIC_90_ (mg/L)
Ceftaroline (0.5–4)	0.0	≤0.5	≤0.5
Clindamycin (0.12–1)	2.0	≤0.12	≤0.12
D-test 1 (NA)	19.4	NA	NA
D-test 2 (NA)	19.4	NA	NA
Daptomycin (0.5–4)	0.0	≤0.5	≤0.5
Erythromycin (0.25–4)	42.9	≤0.25	4.0
Fusidate (0.5–4)	8.2	≤0.5	1.0
Gentamicin (0.25–8)	15.3	0.25	8.0
Levofloxacin (0.5–4)	19.4	≤0.5	4.0
Linezolid (2–16)	0.0	≤2.0	≤2.0
Moxifloxacin (0.25–2)	19.4	≤0.25	2.0
Rifampin (0.03–1)	3.1	≤0.03	≤0.03
Teicoplanin (1–16)	0.0	≤1.0	≤1.0
Telavancin w/tween mimic (0.03–1)	0.0	≤0.03	0.06
Tetracycline (0.5–4)	25.5	≤0.5	4.0
Tobramycin (0.25–8)	27.5	≤0.25	8.0
Trimethoprim/sulfamethoxazole (1/19–8/152)	4.1	1.0	1.0
Vancomycin (0.5–16)	0.0	1.0	1.0

MIC, minimum inhibitory concentration.

### 3.2 Phylogenetic analysis and bacterial typing

We observed 21 different STs among the CA-MRSA isolates, with ST8 being the most prevalent at 34.7%. Additionally, we identified 40 different *spa*-types, of which t008 was the most common, making up 27.6% (Supplementary Figure S1).

The accessory gene regulator (*agr*) typing analysis, a regulatory component involved in the control of bacterial virulence factor expression, showed 58 (59.2%) isolates belonging to *agr* group I, 30 (30.6%) isolates belonging to *agr* group III,9 (9.2%) isolates belonging to *agr* group II and one (1.0%) isolate belonging to *agr* group IV.

#### 3.2.1 cgMLST analysis

**Figure 1:**
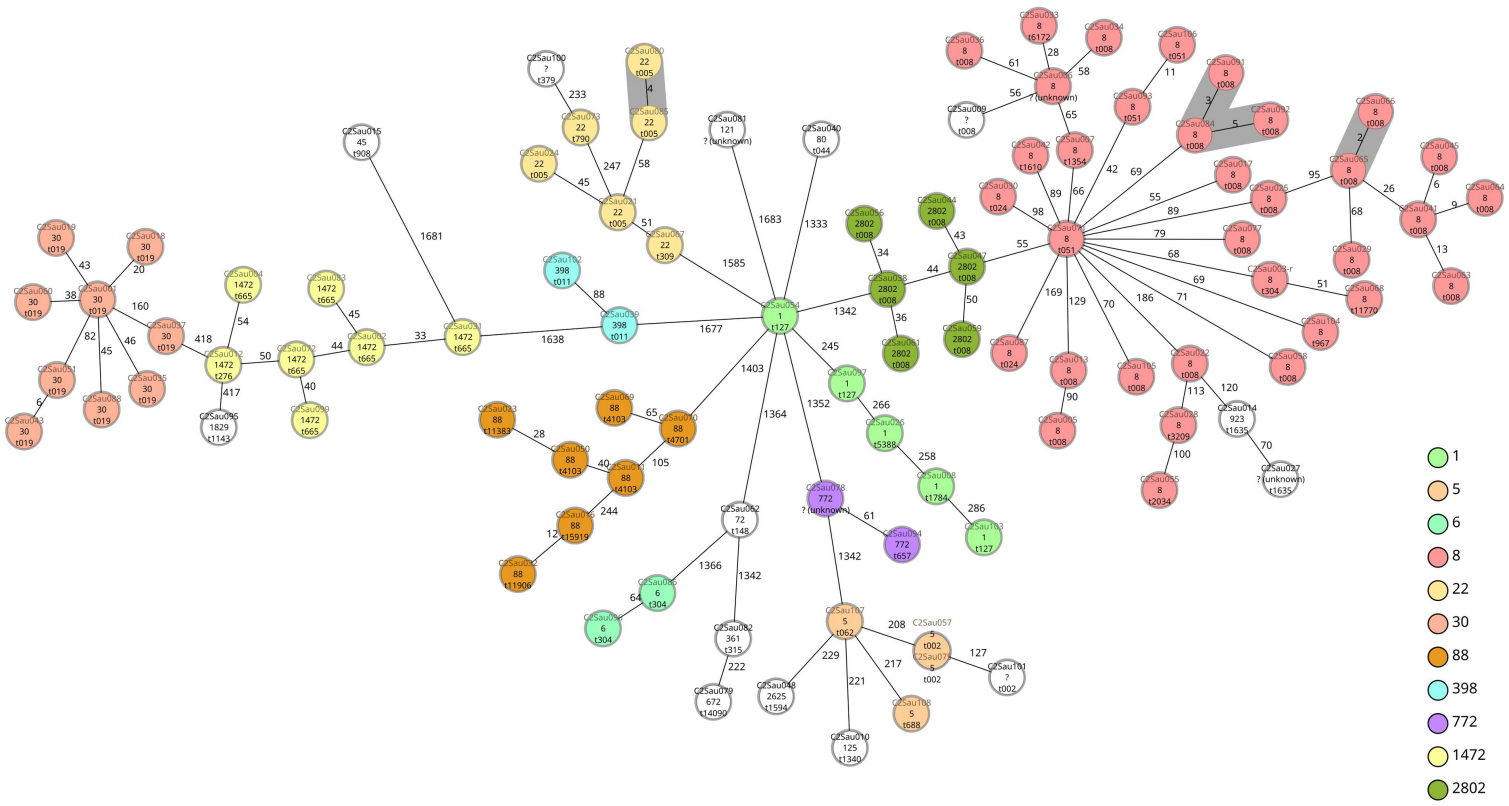
Minimum spanning tree of 98 CA-MRSA isolates causing children infections in Spain based on a cgMLST scheme of 1,861 target genes (SeqSphere+ Ridom, GmbH, Münster, Germany). A threshold of ≤ 5 allelic differences has been applied to highlight in grey shadow genetically related isolates. Isolate names (“C2” refers to study COSACO 2 CA-MRSA infections; “Sau” refers to *Staphylococcus aureus*; followed by internal numbers assigned), STs and *spa*-types are indicated for each isolate. Each circle represents an isolate unless more than one isolate having no allele differences are grouped, in this case two or more different names are displayed. Colors indicate ST and have been used only with STs found more than once (white color indicates STs represented with one unique isolate). Groups observed are (grey shadow): C2Sau080 and C2Sau085 (4 allele differences), t005, ST22; C2Sau084, C2Sau091, C2Sau092 (3–5 allele differences), t008, ST8; C2Sau065 and C2Sau066 (2 allele differences), t008, ST8; C2Sau057 and C2Sau075 (0 allele differences), t002, ST5.

We observed four groups of two to three isolates when applying a ≤ 5 allele differences threshold, having the same antibiotic susceptibility pattern. Two groups were formed by two (C2Sau065 and C2Sau066; 2 allele differences) and three isolates (C2Sau084, C2Sau091 and C2Sau092; between 3-5 allele differences) belonging to ST8/t008, from the same hospital in both cases. One group was formed by two isolates (C2Sau080 and C2Sau085; 4 allele differences) belonging to ST22/t005 from the same hospital, and another group by two ST5/t002 isolates (C2Sau057 and C2Sau075; 0 allele differences) from different hospitals (Figure 1; Supplementary datasheets S1, S2). Isolates belonging to the same group had the same genetic content regarding antibiotic resistance genes and virulence factors, except for one gene in some cases, according to AMRFinderPlus and VFDB results from Ridom SeqSphere+ analysis (Supplementary datasheets S1, S2).

#### 3.2.2 Core-genome SNP-based analysis

Isolates that clustered together by cgMLST analysis, considering a threshold of less than or equal to 5 allele differences, grouped by core-genome SNP analysis as follows: the group formed by ST5/t002 isolates, from different hospitals, did not have core-SNPs; regarding the groups formed by ST8/t008 isolates: one group had six core-SNPs and another group had between 0 and 8 core-SNPs; and the group formed by ST22/t005 had 14 core-SNPs (Supplementary Figure S2).

### 3.3 Analysis of SCC*mec*, antibiotic resistance genes, plasmid replicon genes, and virulence factors

#### 3.3.1 SCC*mec* typing

We observed three main SCC*mec* types in our CA-MRSA isolate collection from pediatric infections: SCC*mec* type IV (2B) (89/98, 90.8%)—SCC*mec* IVc, 51.0%; SCC*mec* IVa, 35.7%; SCC*mec* IVb, 3.1%; SCC*mec* IVh, 1.0%—SCC*mec* type V (5C2) (4/98, 4.1%) and SCC*mec* type V (5C2&5) (4/98, 4.1%) (Table 2). Among SCC*mec* type IV (2B), subtype IVc (50/98, 51.0%) was the most prevalent, and it was associated with ST8 (27/98, 27.6%) and t008 (16/98, 16.3%) isolates. Two ST398 (t011) isolates had the SCC*mec* type V (5C2&5), subtype Vc. One isolate (ST1, t127) had two SCC*mec* elements: SCC*mec* type I (1B) and SCC*mec* type IV (2B), due to the presence of two *ccr* gene complexes: *ccr* class 1 (A1B1) and *ccr* class 2 (A2B2) (Table 2).

**Table 2:** SCC*mec* types, and their associated STs and *spa*-types, found in 98 CA-MRSA isolates causing infections in children in Spain.

SCC*mec* type/Subtype	Number of isolates (%)	ST (*n*)	*spa*-type (*n*)
**SCC*mec* type IV (2B)**	89 (90.8%)		
**IVa**	35 (35.7%)		
		ST88 (7)	t4103 (3), t4701 (1), t11383 (1), t11906 (1), t15919 (1)
		ST1472 (7)	t665 (6), t276 (1)
		ST22 (6)	t005 (4), t309 (1), t790 (1)
		ST8 (3)	t008 (3)
		ST1 (2)	t127 (1), t1784 (1)
		ST5 (2)	t062 (1), t688 (1)
		ST6(2)	t304 (2)
		ST45 (1)	t908 (1)
		ST72 (1)	t148 (1)
		ST923 (1)	t1635 (1)
		ST1829 (1)	t1143 (1)
		ST2625 (1)	t1594 (1)
**IVb**	3 (3.1%)		
		ST8 (2)	t2034 (1), t3209 (1)
		ND (1)	t002 (1)
**IVc** ^**1**^	50 (51.0%)		
		ST8 (27)	t008 (16), t051 (3), t024 (1), t304 (19, t967 (1), t1354 (1), t1610 (1), t6172 (1), 1,176 (1)
			t019 (9)
		ST30 (9)	t008 (6)
		ST2802 (6)	t127 (1), t5388 (1)
		ST1 (2)	t002 (2)
		ST5 (2)	t044 (1)
		ST80 (1)	t1340 (1)
		ST125 (1)	
**IVh**	1 (1.0%)		t379 (1)
		ND (1)	
**SCC*mec* type V (5C2)** ^2^	4 (4.1%)		
		ST772 (2)	t657 (1)
		ST8 (1)	t008 (1)
		ST361 (1)	t315 (1)
**SCC*mec* type V (5C2&5)** ^3^	4 (4.1%)		
**V**	2 (2.0%)		
		ST121 (1)	ND (1)
		ST672 (1)	t14090 (1)
**Vc**	2 (2.0%)		
		ST398 (2)	t011 (2)
**Double**	1 (1.0%)		
**SCC*mec* type I (1B)**			
**SCC*mec* type IV (2B)**			
		ST1 (1)	t127 (1)

The *Ccr* gene complex and the *mec* gene complex are indicated in brackets next to the SCC*mec* type.

1 One isolate had 52.77% coverage with the SCC*mec* cassette template.

2 Two isolates had 57.52 and 54.40% coverage with the *SCCmec* cassette template.

3 One isolate had 64.08% coverage with the *SCCmec* cassette template.

#### 3.3.2 Antibiotic resistance genes

All isolates had the *mecA* gene (Table 3). RFPlasmid analysis evenly predicted this gene in plasmid (47%) and chromosomal contigs (53%) (Supplementary Table S2). SSC*mec* is a mobile genetic element sharing genetic features with plasmids, such as insertion sequences and transposons, which could explain the classification of those contigs as plasmids (Malachowa and DeLeo, 2010). Furthermore, no plasmid replicon genes were found in the same contigs as the *mecA* genes. Regarding resistance to aminoglycosides, the *aac(6′)-Ie/aph(2″)-Ia* gene, encoding a two-domain acetyltransferase/phosphotransferase enzyme and related to gentamicin and tobramycin resistance, was detected in 15 isolates; and the *aadD1* gene encoding a nucleotidyltransferase, related to tobramycin resistance, was found in 15 isolates. All 27 tobramycin-resistant isolates had at least one of these genes (Table 3). All *aac(6′)-Ie/aph(2″)-Ia* genes were predicted in a plasmid contig, whereas all *aadD1* genes were predicted in a chromosomal contig (Supplementary Table S2). The majority of the levofloxacin and moxifloxacin-resistant isolates had point mutations in *parC* (*n* = 23; S80F, S80Y), *gyrA* (*n* = 18; S84L, S85P), or *parE* genes (*n* = 1, P451S). Fusidic acid resistance could be explained by the presence of *fusC* (*n* = 6), *fusB* (*n* = 1) and *fusA* (*n* = 1) genes, mainly predicted in plasmid contigs (Supplementary Table S2). The phenotype of erythromycin resistance and clindamycin resistance (constitutive or inducible) could be explained by the presence of *erm(C)* (*n* = 17). The phenotype of erythromycin resistance but susceptible clindamycin could be explained by the *msr*(A) (*n* = 21) and *mph*(C) (*n* = 19) genes; 19.4% (*n* = 19) of isolates had both genes. Clindamycin resistance gene *lnu(A)* was detected in three isolates. All these macrolides and/or lincosamides resistance genes were predicted in plasmid contigs. Tetracycline resistance could be explained by the presence of *tet(K)* (*n* = 21), *tet(L)* (*n* = 3), and *tet(M)* (*n* = 2), mainly predicted in plasmid contigs (Table 3 and Supplementary Table S2).

**Table 3:** Concordance between antibiotic resistance phenotypes and genotypes based on antibiotic resistance genes (ARGs) and point mutations, in CA-MRSA isolates (n_t_ = 98) causing infections in children in Spain.

Antibiotic family	Antibiotic tested	Phenotype	CA-MRSA *n* (%)	Genotype^a^ (ARGs and point mutations)				ARG (*n*)
				Presence		Absence		
				*n*	(%)	*n*	(%)	
Aminoglycosides	Gentamicin	Resistant	15 (15.3)	15	(100.0)	0	(0.0)	*aac(6′)-Ie/aph(2″)-Ia* (15)
		Susceptible	83 (84.7)	0	(0.0)	83	(100)	
	Tobramycin	Resistant	27 (27.5)	26	(96.3)	1	(3.7)	*aac(6′)-Ie/aph(2″)-Ia* (15)*, aadD1* (15)^b^
		Susceptible	71 (72.4)	1	(1.4)	70	(98.6)	
β-Lactams (second-generation cephalosporins)	Cefoxitin	Resistant	98 (100)	98	(100.0)	0	(0.0)	*mecA* (98)
		Susceptible	0 (0.0)	–	–	–	–	
Fluoroquinolones	Levofloxacin, moxifloxacin	Resistant	20^c^ (20.4)	18	(90.0)	2	(10.0)	*gyrA* (18), *parC* (23), *parE* (1) mutations^e^
		Not resistant^d^/Susceptible	78 (79.6)	6	(7.7)	72	(92.3)	
Miscellaneus agents^f^	Fusidic acid	Resistant	8 (8.2)	8	(100.0)	0	(0.0)	*fusA(1), fusB* (1), *fusC* (6)
		Susceptible	90 (91.8)	0	(0.0)	90	(100)	
Lincosamides	Clindamycin	Resistant (constitutive or inducible)	20^g^ (20.4)	17	(85.0)	3	(15.0)	*lnu(A)* (3) *erm(C)* (17)
		Susceptible	78 (79.6)	3	(3.8)	75	(96.2)	
Macrolides	Erythromycin	Resistant	42 (42.9)	38	(90.5)	4	(9.5)	*erm(C)* (17), *mph(C)* (19), *msr(A)* (21)^h^
		Susceptible	56 (57.1)	0	(0.0)	56	(100)	
Tetracyclines	Tetracycline	Resistant	25 (25.5)	23	(92.0)	2	(12.0)	*tet(K)* (21), *tet(L)* (3), *tet(M)* (2)^i^
		Susceptible	73 (74.5)	0	(0.0)	73	(100)	

All ARGs detected had a percentage of identity above 90% and a coverage above 85%. In some isolates, we detected ARGs in trimmed reads but not in the genome assemblies: *erm(C)*, *n* = 3; *fusA*, *n* = 1; *tet(K)*, *n* = 1.

a We indicate presence (+) or absence (−) of ARGs related to the resistance phenotype.

b Three isolates had both *aac(6′)-Ie/aph(2″)-Ia* and *aadD1* genes.

c Eighteen isolates were resistant to both levofloxacin and moxifloxacin, one isolate was resistant to levofloxacin only, and one isolate was resistant to moxifloxacin only.

d Non-resistant indicates levofloxacin MICs ≤ 0.5 mg/L (lowest range in microdilution panel), whereas EUCAST breakpoints consider susceptible a MIC ≤0.001 and resistant a MIC >1 mg/L.

e Eighteen isolates had mutations in both *gyrA* and *parC* genes.

f Classification according to the EUCAST.

g Eighteen isolates had inducible resistance, one isolate had constitutive resistance, and one isolate had both constitutive and inducible resistance.

h Nineteen isolates had both *mph(C)* and *msr(A)* genes.

i One isolate had both *tet(k)* and *tet(M)* genes; one isolate had both *tet(L)* and *tet(M)* genes.

The *aac(6′)-Ie/aph(2″)-Ia* and *erm(C)* genes were present in 83.3 and 66.7% of ST22 isolates, respectively. The *mph(C)* and *msr*(A) genes were present in 75% of ST5 isolates and 100% of ST1492 isolates. The *tet(k)* gene was found in 60% of ST1 isolates (Supplementary Figure S3).

#### 3.3.3 Plasmid replicon genes

96% (*n* = 94) of the isolates had one or more replicon genes and 58% had between 2 and 3 different replicon genes (Supplementary Table S3). The most prevalent replicon types were *rep7c* (52.0%), which belongs to the Rep_trans family, a Rolling-Circle Replicating (RCR) plasmid type, and *rep20* (50.0%), which belongs to the Rep_1 family, a RCR plasmid type. These two replicon types were found in 15 isolates (15.3%) (Supplementary Table S3).

Plasmid replicon *rep7c* gene was found in all ST1, ST8, and ST2802 isolates; plasmid replicon *rep20* gene was found in more than 85% of ST5 and ST8 isolates; plasmid replicon *rep5a* gene was found in all ST1—together with *rep7c*; plasmid replicon *rep16*, *rep19*, and *rep21*genes were found in 90, 90, and 100% of ST30 isolates, respectively; plasmid replicon *rep7a* gene was found in all ST398 and ST1472 (Supplementary Figure S5).

When analyzing plasmid replicon genes and ARGs detected in the same contig, *blaZ*, *mph(C)*, and *msr(A)* were associated with different plasmid replicon types: mainly *rep16*, *rep19*, and *rep20,* whereas *erm(C)*, *lnu(A)*, and *tet(K)* were associated with unique plasmid replicon types: *rep10*, *rep13*, and *rep7a*, respectively (Supplementary Figure S4).

#### 3.3.4 Virulence factors

Virulence factors related to adhesion, *capA* and *capP* genes, to lipid degradation, *geh* gene, to hemolysis, *hlb*, *hld*, *hlgABC*, and *hly/hla* genes, and to tissue destruction, *sspAB* genes, were found in all CA-MRSA isolates. In addition, *dltABCD* genes, involved in surface charge modifications, and *arlRS* genes, involved in the regulation of adhesion and autolysis, were present in all CA-MRSA isolates (Table 4 and Supplementary Figure S6). All isolates but one ST398 had *scn* gene, involved in immune evasion. *LukF-PV* and *lukS-PV* genes, encoding Panton-Valentine leukocidin (PVL), were found in 75.5% of isolates, being present in all ST30, ST88, ST1472, and ST2802 isolates and 94.1% of ST8 isolates. Among PVL-positive CA-MRSA isolates (*n* = 92), 56.5% were *agr* type I, 32.6% *agr* type III, 9.8% *agr* type II, and 1.1% *agr* type IV, and all PVL-negative CA-MRSA isolates (*n* = 6) were *agr* type I. *Tsst-1* gene, encoding the toxic shock syndrome toxin 1, was found in 10.2% of isolates, being present in 83.3% of ST22 isolates. *Sea*, *seb*, and *sec* enterotoxin genes were found in 8.2, 3.1, and 12.2% of isolates, respectively. ACME-related genes were found in two ST8 (t008) isolates. Copper resistance genes, *copB* and *mco*, were found in two isolates, ST22 (t790) and ST1829 (t1143), whereas mercury resistance genes, *merABR*, were found in 47% of isolates: all ST1472 and ST2802 isolates, 71.4% of ST88 isolates, and 70.6% of ST8 isolates.

**Table 4:** Summary of virulence factors and antiseptic or heavy metal resistance genes found in CA-MRSA isolates causing infections in children in Spain, and their predicted location in plasmid or chromosomal contigs based on RFPlasmid analysis.

Virulence gene	CA-MRSA with the gene (n_t_ = 98), *n* (%)	Gene product	Function	Location		Gene within prophage sequence			
				In chromosome contig, *n* (%)	In plasmid contig, *n* (%)	Yes		No	
						*n*	(%)	*n*	(%)
*aur*	78 (79.6)	Aureolysin	Tissue destruction	78 (100.0)	–	0	(0.0)	78	(100.0)
*capA*	98 (100.0)	Fibrinogen binding proteins	Adhesion	98 (100.0)	–	0	(0.0)	98	(100.0)
*capP*	98 (100.0)			98 (100.0)	–	0	(0.0)	98	(100.0)
*chp*	77 (78.6)	Chemotaxis inhibitory protein	Immune evasion	66 (85.7)	11 (14.3)	74	(96.1)	3	(3.9)
*clfA*	59 (60.2)	Fibrinogen binding proteins	Adhesion	59 (100.0)	–	0	(0.0)	59	(100.0)
*clfB*	60 (61.2)			60 (100.0)	–	0	(0.0)	60	(100.0)
*coa*	8 (8.2)	Staphylocoagulase	Coagulation	8 (100.0)	–	0	(0.0)	8	(100.0)
*eta*	3 (3.1)	Exfoliative toxin A	Scalded skin syndrome	3 (100.0)	–	3	(100.0)	0	(0.0)
*etb*	1 (1.0)	Exfoliative toxin B		–	1 (100.0)	0	(0.0)	1	(100.0)
*geh*	98 (100.0)	Lipase	Lipid degradation	100.0 (98)	–	0	(0.0)	98	(100.0
*hlb*	98 (100.0)	β-Hemolysin	Hemolysis	91 (92.8)	7 (7.1)	78	(79.6)	20	(20.4)
*hld*	98 (100.0)	δ-Hemolysin		98 (100.0)	–	0	(0.0)	98	(100.0)
*hlgA*	98 (100.0)	γ-Hemolysin components		98 (100.0)	–	0	(0.0)	98	(100.0)
*hlgB*	98 (100.0)			98 (100.0)	–	0	(0.0)	98	(100.0)
*hlgC*	97 (99.0)			97 (100.0)		0	(0.0)	97	(100.0)
*hly/hla*	98 (100.0)	α-Hemolysin		96 (98.0)	2 (2.0)	0	(0.0)	98	(100.0)
*hysA*	95 (97.0)	Hyaluronidase	Tissue invasion	95 (100.0)	-	0	(0.0)	95	(100.0)
*lukF-PV*	74 (75.5)	Panton–Valentine leukocidin	Leukotoxin	65 (87.8)	9 (12.2)	66	(89.2)	8	(10.89)
*lukS-PV*	74 (75.5)			65 (87.8)	9 (12.2)	66	(89.2)	8	(10.8)
*sak*	94 (96.0)	Staphylokinase	Clot dissolution	83 (88.3)	11 (11.7)	91	(96.8)	3	(3.2)
*scn*	97 (99.0)	Complement system inhibitory protein	Immune evasion	84 (86.6)	13 (13.4)	90	(92.8)	7	(7.2)
*sea*	8 (8.2)	Enterotoxin A	Food poisoning	8 (100.0)	–	8	(100.0)	0	(0.0)
*seb*	3 (3.1)	Enterotoxin B		3 (100.0)	–	2	(66.7)	1	(33.3)
*sec*	12 (12.2)	Enterotoxin C		11 (91.7)	1 (8.3)	4	(33.3)	8	(66.7)
*spa*	85 (86.7)	Immunoglobulin G-binding protein A	Immune evasion	85 (100.0)	–	0	(0.0)	85	(100.0)
*sspA*	98 (100.0)	Serine protease	Tissue destruction	98 (100.0)	–	No prophage sequence in the same contig			
*sspB*	98 (100.0)	Cysteine protease		98 (100.0)	–	0	(0.0)	98	(100.0)
*tsst-1*	10 (10.2)	Toxic shock syndrome toxin 1	Superantigen	5 (50.0)	5 (50.0)	6	(60.0)	4	(40.0)
*vWbp*	49 (50.0)	Staphylocoagulase	Coagulation	49 (100.0)	–	0	(0.0)	49	(100.0)
Accessory gene regulatory system (agrABCD)^a^									
*agrA*	98 (100.0)	agrA response regulator	Dissemination during acute infection, colonization, and persistence	98 (100.0)	–	No prophage sequence in the same contig			
*agrB*	59 (60.2)	agrB putative AIP processing-secretion protein		59 (100.0)	–	No prophage sequence in the same contig			
*agrC*	52 (53.1)	agrC receptor histidine kinase		52 (100.0)	–	No prophage sequence in the same contig			
*agrD*	1 (1.0)	agrD Agr autoinducing peptide precursor		1 (100.0)	–	No prophage sequence in the same contig			
Arginine catabolic mobile element (ACME)^b^									
*arcA*	2 (2.0)	Arginine deiminase arcA	Colonization of skin and mucous membranes	–	2 (100.0)	No prophage sequence in the same contig			
*arcB*	2 (2.0)	Ornithine carbamoyltransferase arcB		–	2 (100.0)	No prophage sequence in the same contig			
*arcC*	2 (2.0)	Carbamate kinase arcC		–	2 (100.0)	No prophage sequence in the same contig			
*arcD*	2 (2.0)	Arginine/Ornithine antiporter arcD		–	2 (100.0)	No prophage sequence in the same contig			
*argR*	2 (2.0)	Arginine repressor argR		–	2 (100.0)	No prophage sequence in the same contig			
Copper and mercury resistance (COMER) mobile element^c^									
*copB*	2 (2.0)	Copper-translocating P-type ATPase copB	Immune evasion	–	2 (100.0)	No prophage sequence in the same contig			
*mco*	2 (2.0)	Multi-copper oxidase mco		–	2 (100.0)	No prophage sequence in the same contig			
*merA*	46 (47.0)	Mercury (II) reductase merA		33 (71.7)	13 (28.3)	No prophage sequence in the same contig			
*merB*	46 (47.0)	Organomercurial lyase merB		33 (71.7)	13 (28.3)	No prophage sequence in the same contig			
*merR*	45 (47.0)	Regulatory protein merR		33 (73.3)	12 (26.7)	No prophage sequence in the same contig			
dltABCD Operon									
*dltA*	98 (100.0)	Proteins contributing a net positive charge to the *Staphylococcus aureus* surface	Surface charge modifications	98 (100.0)	–	0	(0.0)	98	(100.0)
*dltB*	98 (100.0)			98 (100.0)	–	0	(0.0)	98	(100.0)
*dltC*	98 (100.0)			98 (100.0)	–	0	(0.0)	98	(100.0)
*dltD*	98 (100.0)			98 (100.0)	–	0	(0.0)	98	(100.0)
Regulatory system arlRS									
*arlR*	98 (100.0)	Histidine-protein kinase	Regulation of adhesion, autolysis, multidrug resistance, and virulence	98 (100.0)	–	0	(0.0)	98	(100.0)
*arlS*	98 (100.0)	Putative response regulator ArlR		98 (100.0)	–	0	(0.0)	98	(100.0)

a One isolate had all agrABCD genes and was found together in the same contig.

b Genes *arcA*, *arcB*, *arcC*, *arcD*, and *argR* were found together in the same contig in each isolate.

c Genes *copB* and *mco* were found together in the same contig in each isolate; genes *merA*, *merB*, and *merR* were found together in the same contig in each isolate (one isolate lacked the *merR* gene).

### 3.4 Bacteriophage detection

All isolates had one or more phage sequences with more than 90% of *Staphylococcus* spp. identity, 13 isolates also had phage sequences with >90% of *Streptococcus* spp. identity in different contigs as for the *Staphylococcus* spp. phage sequences. *Streptococcus* spp. phage sequences were generally shorter than those of *Staphylococcus* spp. phage sequences, 4,600 bp, whereas *Staphylococcus* spp. phage sequences ranged from 2,338 bp to 514,137 bp.

All *eta* (3/3, 100%) and *sea* genes (8/8, 100%) were found in prophage sequences, and also the majority of *chp* (74/77, 96.1%), *hlb* (78/98, 79.6%), *sak* (91/94, 96.8%), *scn* (90/97, 92.8%), *lukFS-PV* (66/74, 89.2%), *seb* (2/3, 66.7%), and *tsst-1* (4/10, 60%) genes (Table 4).

### 3.5 Comparative analysis with CA-MRSA isolates from nasal colonization in children

Among previously studied CA-MRSA isolates from nasal colonization in children, 47.4% were resistant to levofloxacin and moxifloxacin, 21% were resistant to erythromycin, 15.8% were resistant to tobramycin, and 5.3% were resistant to fusidic acid. All CA-MRSA isolates from nasal colonization were negative for PVL encoding genes, and 21.1% were positive for the *tsst-1* gene. Common STs between CA-MRSA isolates causing infections in children and CA-MRSA isolates from nasal colonization were: ST5, ST6, ST22, ST30, ST72, and ST125; nevertheless, no genetic relatedness was found using cgMLST analysis (≤5 alleles by cgMLST) (Supplementary Figures S7, S8). All isolates from ST5 (four from infections and six from nasal colonizations) had *fosB* and *tet(38)* genes but differed in the presence of aminoglycoside resistance genes (i.e., *aph(3′)-IIIa*) and erythromycin resistance genes [i.e., *mph(C)/msr(A)*]. In this case, t002 was the most prevalent *spa*-type in both isolate collections. On the contrary, ST30 isolates (nine from infections and four from nasal colonizations) differed in the *spa*-types, with t019 being the only *spa*-type among isolates from infections and t012 the most prevalent *spa*-type among isolates from nasal colonization. All ST30 isolates from infections had PVL genes. Isolates from ST22 (6 from infections and two from nasal colonizations) differed in the *spa*-types and antibiotic resistance genes. ST22 isolates from infections had aminoglycoside (i.e., *aac(6′)-Ie/aph(2″)-Ia*), erythromycin [i.e., *erm(C)*], trimethoprim (i.e., *dfrC*) resistance genes, and mutations in quinolone targets (i.e., *gyrA*_S84L / *parC*_S80F) that were not present in ST22 isolates from nasal colonizations. In addition, 66.7% (4/6) ST22 isolates from infections had PVL genes (Supplementary datasheets S1, S2).

## 4 Discussion

Ninety-eight CA-MRSA isolates from pediatric infections, mostly SSTIs, in Spain were analyzed using WGS in this study. ST8, t008, and SCC*mec* type IV (2B) were the most common lineages, but still, the number of different STs found in this collection highlights the diversity of CA-MRSA isolates able to cause infections among children. The ST8-SCC*mec* IVc lineage was previously described as the most common in Spain in a study during 2004–2012, including the children and adult population (Vindel et al., 2014). Other common lineages within this collection, such as the Southwest Pacific ST30-IVc clone and the African ST88-IVa clone, have also been previously reported in Spain (Vindel et al., 2014) and other countries (Otter and French, 2010; Breurec et al., 2011). The ST1472-IVa clone has been previously described in Spain but associated with methicillin-susceptible *S. aureus* (MSSA) (Gasch et al., 2012). Two ST398-Vc isolates, an originally livestock-associated clade, were identified in this collection; however, one of these isolates was positive for *scn* virulence gene—a human-specific immune evasion genetic marker (Zhao et al., 2020)—and negative for *tet(M)* tetracycline resistance gene—a genetic marker of the pig-associated clade (Price et al., 2012)—what indicates human-adapted ST398 MRSA (Laumay et al., 2021). Recently, an increase in macrolide resistance in MSSA isolates from blood has been described in Spain, associated with the spreading of ST398/*erm*T isolates, 78.6% of them had the *scn* virulence gene, and 14.3% had tetracycline resistance *tet(M)* gene (El Mammery et al., 2023).

In contrast, finding clusters of genetically related CA-MRSA isolates (≤5 allele differences by cgMLST and 0–6 core-genome SNPs), within the same hospital and from geographically separated hospitals, suggests a possible common origin and patient-to-patient transmission of CA-MRSA. A study about household transmission of USA300 CA-MRSA in the United States using WGS demonstrated that single strains were transmitted within households and could persist for 2.5–8.5 years (Alam et al., 2015). In another study, an agent-based model was used to represent population behavior, locations, and contact patterns using CA-MRSA data from Chicago, IL, USA, which indicated that contact with colonized individuals in households was probably the primary source of CA-MRSA acquisition. The authors suggested that interrupting household transmission should be the main target to control CA-MRSA (Macal et al., 2014). Because of this scenario, in which an MRSA spreads to multiple members of the patient’s household or community, Salgado et al. suggested a different term, “community-onset,” to describe the patient’s location at the time of identification of MRSA and to avoid a wrong origin assignment (Salgado et al., 2003).

Approximately half of the CA-MRSA isolates in this study were additionally resistant to erythromycin, and approximately one-third were additionally resistant to tobramycin and tetracycline. Classically, CA-MRSA is usually susceptible to the majority of antibiotics other than methicillin and *β*-lactams, while multi-resistance is common in HA-MRSA isolates (Otto, 2013). The increase in macrolide consumption in Spain, mainly outpatient use of azithromycin, has been related to the increase in macrolide resistance in MSSA isolates from blood (El Mammery et al., 2023), and could also explain the high percentage of co-resistance to erythromycin in CA-MRSA in this study. Erythromycin resistance could be explained mainly by the plasmid-located *erm(C)*, *mph(C)*, and *msr(A)* genes. Both *mph(C)* and *msr(A)* genes were mainly found together and in the same contig; this association has been previously described (El Mammery et al., 2023). Tetracyclines are a possible treatment option for patients with community-onset MRSA SSTI. In this study, the plasmid-located *tetK* gene was the primary mechanism of tetracycline resistance, and the plasmid-located *tetL* and the chromosomal or transposonal *tetM* genes were rarely detected. The *tetM* gene (76%) followed by the *tetK* gene (73%) were vastly present in MRSA isolates in the European SENTRY program published in 2001, whereas *tetK* gene was the most common in MSSA isolates (Schmitz, 2001). In a recent study on MRSA CC398 from Spanish hospitals, all tetracycline-resistant isolates carried the *tetM* gene, and 75% of them carried the *tetK* gene as well (Ceballos et al., 2020). In this collection of CA-MRSA isolates causing infections in children, almost all isolates (96%) had a plasmid replicon gene, RCR plasmid type (84.7%) being slightly more prevalent than the tetha-replicating plasmid type (74.5%), whereas in a previous study analyzing 278 non-identical *S. aureus* plasmids, theta-type mechanism was more prevalent (57%) (Kwong et al., 2017). In both studies, the prevalence trend for RCR initiator types was Rep_trans > Rep_1 > RepL. Still, for tetha-replication plasmids, those using Rep_3 initiator were the highest in our study compared to RepA_N initiators in the study by Kwong et al. (2017).

The success of CA-MRSA causing infections is partly due to their enhanced virulence (Otto, 2013; Otto, 2010), such as via the acquisition of the phage-encoded PVL genes, which were highly present (75%) in this collection, as previously described in CA-MRSA isolates (Boyle-Vavra and Daum, 2007; DeLeo et al., 2010). The successful spread of USA300 CA-MRSA was related to genes present in the ACME. Only two (2%) ST8-SCC*mec* IVa isolates were ACME-positive in this study, representing the USA300 clone, since this element is limited to strain USA300 (Otto, 2013). A study on CA-MRSA in adults in Spain between 2004 and 2012 showed 8.9% of the USA300 clone (ST8-IVa-ACME-positive) (Vindel et al., 2014). The evolution of CA-MRSA is related to the combination of different events, on one side, the acquisition of the SSC*mec* type IV, which is shorter and thus believed to cause less of a fitness burden, on the other side, an increased toxin expression and the acquisition of novel virulence genes on mobile genetic elements (MGE) (Otto, 2013; Otto, 2010). In this regard, all *eta* (exfoliative toxin A) and *sea* genes (enterotoxin A) were phage-located in this study as previously described (Turner et al., 2019). In contrast, the ɑ-hemolysin (Hla, ɑ-toxin) encoding gene, a cytolysin with pro-inflammatory effects that is produced by most *S. aureus* strains (Otto, 2013), was present in all CA-MRSA isolates in this collection, in addition to other cytolytic and pore-forming proteins, such as *γ*-hemolysin (HlgAB, HlgCB) and *δ*-hemolysin (δ-toxin). CA-MRSA strains commonly have high Agr activity, a regulator that may contribute to the increased expression of virulence factors (Otto, 2010). In this study, *agr* type I was the most prevalent as previously described in a Spanish study during the period 2004–2012 (Vindel et al., 2014), but *agr* type III is higher in the present study (32.6%) than before (10.2%) (Vindel et al., 2014).

This study has some limitations. Despite being one of the most extensive series to date on WGS-based typing of CA-MRSA causing infections in children, the sample size may not be sufficient to generalize the results to other regions or contexts. Although this is a multicenter study, our findings are limited to the population covered by those centers included in the study. The retrospective design, based on reviewing hospital records that were not standardized for this study, may have limited our findings. A more extensive prospective study at a national level, including all country regions, similar to the nationwide surveillance study on CA-MRSA nasal colonization in Spanish children (Del Rosal et al., 2020), would improve our knowledge on the molecular epidemiology and characteristics of MRSA causing infections in children. Furthermore, collecting epidemiological data on contacts, including households as important reservoirs, could help us understand the dynamics of *S aureus* acquisition and transmission, especially in SSTI among children.

Nasal colonization with MRSA has been correlated with a higher chance of MRSA infections (Von Eiff et al., 2001; Wertheim et al., 2005); nevertheless, colonization is mainly due to MSSA as described in individuals in the USA (Otto, 2010) and in Spanish children (Román et al., 2021), and only 1.5% of MRSA nasal carriage was found among individuals in the United States (Otto, 2010) and 1.4% among Spanish children (Román et al., 2021). Interestingly, when we comparatively analyzed previous CA-MRSA from nasal colonization in Spanish children (Letunic and Bork, 2021) with CA-MRSA isolates causing infections included in this study, we observed differences in the lineages and presence of virulence factors, with t002 and ST5 being the most prevalent in nasal carriers, with no PVL-positive isolates. Although both studies have been carried out at different time and in different populations and therefore comparisons should be carefully taken, these findings rise the possibility that infection in the absence of colonization could be possible, such as by direct body contact with infected individuals or contaminated fomites, and that even other body sites than the nostrils, such as throat, axilla, groin and perirectal area could be source of MRSA colonization (DeLeo et al., 2010).

## 5 Conclusion

In summary, we described a diverse collection of CA-MRSA isolates causing infections in children, mostly SSTIs, with ST8-IVc as the most prevalent lineage. The phylogenetic analysis results suggested possible contact transmission due to the presence of genetically related ST8-IVc isolates from the same hospital, but also possible common origin of ST5-IVc isolates from different hospitals and different geographic areas. A correlation between nasal colonization and infection has been accepted in the epidemiology and etiology of CA-MRSA isolates. However, we observed different lineages among CA-MRSA isolates causing infections and CA-MRSA isolates from nasal colonization in Spanish children. Future studies on CA-MRSA isolates causing infections and their colonization rate in the same population should be performed to better understand the extent to which MRSA colonization is involved in the development of CA-MRSA infections. Finally, this study represents a first step toward implementing WGS in CA-MRSA surveillance in Spain, following the roadmap set by the ECDC at the European level.

## Data Availability

The data that support the findings of this study are openly available in figshare: García-Cobos, Silvia (2024). Virulence genes (VFDB). figshare. Dataset. https://figshare.com/articles/dataset/Virulence_genes_VFDB_/25298683. García-Cobos, Silvia (2024). Virulence genes (ACME). figshare. Dataset. https://figshare.com/articles/dataset/Virulence_genes_ACME_/25298692. García-Cobos, Silvia (2024). Virulence factors (COMER). figshare. Dataset. https://figshare.com/articles/dataset/Virulence_factors_COMER_/25298713. García-Cobos, Silvia (2024). Virulence genes (in-house database). figshare. Dataset. https://figshare.com/articles/dataset/Virulence_genes_in-house_database_/25298725. García-Cobos, Silvia (2024). Agr typing. figshare. Dataset. https://figshare.com/articles/dataset/Agr_typing/25298734. García-Cobos, Silvia (2024). Assembly report (quast v5.0.2). figshare. Dataset. https://doi.org/10.6084/m9.figshare.25298749.v1. García-Cobos, Silvia (2024). ST-MLST, *spa*-types, cgMLST and NCBI AMRFinderPlus (SeqSphere+ v 9.0). figshare. Dataset. https://doi.org/10.6084/m9.figshare.25298809.v1. García-Cobos, Silvia (2024). RFPlasmid results. figshare. Dataset. https://doi.org/10.6084/m9.figshare.25298830.v1. García-Cobos, Silvia (2024). Distance matrix core genome SNP-based analysis. figshare. Dataset. https://doi.org/10.6084/m9.figshare.25298845.v1. García-Cobos, Silvia (2024). Prophage sequence detection (DBSCAN-SWA). figshare. Dataset. https://doi.org/10.6084/m9.figshare.25298857.v1. García-Cobos, Silvia (2024). SSCmec typing. figshare. Dataset. https://doi.org/10.6084/m9.figshare.25298872.v1. García-Cobos, Silvia (2024). Plasmid replicon genes and types. figshare. Dataset. https://doi.org/10.6084/m9.figshare.25289410.v1. García-Cobos, Silvia (2024). Antibiotic resistance genes. figshare. Dataset. https://doi.org/10.6084/m9.figshare.25289347.v1. García-Cobos, Silvia (2024). Antibiotic Resistance Genes (ARGANNOT) (trimmed reads screening). figshare. Dataset. https://doi.org/10.6084/m9.figshare.25471111.v1. García-Cobos, Silvia (2024). Antibiotic Resistance Genes (CARD) (trimmed reads screening). figshare. Dataset. https://doi.org/10.6084/m9.figshare.25470946.v1. García-Cobos, Silvia (2024). Antibiotic Resistance Genes (CARD) (trimmed reads screening). figshare. Dataset. https://doi.org/10.6084/m9.figshare.25470313.v1.

## Footnotes

^1^ https://github.com/rrwick/Unicycler

^2^ https://github.com/ablab/quast

^3^ https://www.ridom.de/seqsphere/

^4^ https://www.cgmlst.org/ncs/schema/141106/

^5^ https://github.com/tseemann/snippy

^6^ https://github.com/amkozlov/raxml-ng

^7^ https://bactopia.github.io/v3.0.0/bactopia-tools/staphtyper/

^8^ https://cge.food.dtu.dk/services/SCCmecFinder-1.2/

^9^ https://github.com/tseemann/abricate

^10^ http://genepi.food.dtu.dk/resfinder

^11^ https://www.mgc.ac.cn/VFs/

^12^ https://cge.food.dtu.dk/services/PlasmidFinder/

^13^ https://github.com/sanger-pathogens/ariba

^14^ https://www.mediterranee-infection.com/acces-ressources/base-de-donnees/arg-annot-2/

^15^ https://card.mcmaster.ca/

^16^ https://github.com/aldertzomer/RFPlasmid

^17^ https://github.com/HIT-ImmunologyLab/DBSCAN-SWA
